# A Case of Malignant Pleural Effusion Complicated by Klebsiella rhinoscleromatis

**DOI:** 10.7759/cureus.71520

**Published:** 2024-10-15

**Authors:** Diana Brites, Catarina Forra, Mafalda Ferreira, Sara Sintra, Maria Eugénia André

**Affiliations:** 1 Internal Medicine, Unidade Local de Saúde de Castelo Branco, Castelo Branco, PRT

**Keywords:** complicated pneumonia, klebsiella pneumoniae, klebsiella rhinoscleromatis, malignant pleural effusion, pleural bacterial infection

## Abstract

This case report describes an elderly man with a history of alcohol use disorder and primary malignant liver neoplasm who presented to the emergency department with dyspnea, asthenia, fever, and signs of respiratory distress. Diagnostic tests, including a chest radiograph and computed tomography, revealed a large left-sided exudative pleural effusion with regular and diffuse pleural enhancement, a large tumor lesion in the left lobe with diaphragmatic and pleural invasion, and a liver with features suggestive of cirrhosis. Laboratory data showed increased inflammatory markers and hyperlactacidemia. Following thoracentesis, pleural fluid culture revealed the growth of *Klebsiella rhinoscleromatis*. Despite targeted antibiotic therapy with the resolution of the infectious condition, the patient's clinical condition worsened, resulting in multiorgan failure, and the patient ultimately died in this context.

## Introduction

*Klebsiella pneumoniae* is a gram-negative bacterium that belongs to the *Enterobacteriaceae* family, with its virulence depending on various factors [1-3]. It is considered to be part of a group of highly virulent microorganisms capable of developing antibiotic resistance [2-4]. It is most frequently associated with alcohol use disorder and diabetes mellitus [3], which are the main risk factors. This bacterium typically colonizes the oropharyngeal and gastrointestinal tracts, making it a common cause of pneumonia and urinary tract infections, particularly as a nosocomial infection [1,4], but it is also increasingly found in community-acquired infections. Humans are the primary reservoir, and colonization among hospitalized individuals is more frequent [1]. *Klebsiella pneumoniae* is responsible for approximately 3%-5% of community-acquired pneumonia in the West and 11% worldwide, with a high mortality rate [1].

*Klebsiella pneumoniae* comprises three subspecies: the *pneumoniae* subspecies is responsible for lower respiratory tract infections, while the *ozaenae* and *rhinoscleromatis* subspecies are associated with upper respiratory tract infections, specifically atrophic rhinitis and rhinoscleroma, respectively [4]. *Klebsiella pneumoniae* subspecies *rhinoscleromatis* is almost exclusively associated with infections of the nasal cavity [5], and infection that affects the pleural fluid has not been reported to date. Thus, for the first time, a case is described of an elderly man with large malignant pleural effusion and microbiological isolation of *Klebsiella rhinoscleromatis* in the pleural fluid culture, a microorganism that rarely causes such lower respiratory tract conditions.

## Case presentation

A man in his 70s presented to the emergency department with dyspnea and asthenia lasting for over two weeks. The patient had a past medical history of hypertension and atrial fibrillation, for which he was on chronic medication, alcohol use disorder, and a recent diagnosis of a large hepatocellular carcinoma in the cirrhotic liver, approximately 19 cm in diameter. Upon hospital admission, his blood pressure was stable (116-120/62-72 mmHg), his heart rate was slightly elevated (110-115 beats per minute), and he presented with tachypnea (respiratory rate of 24 breaths per minute) with peripheral oxygen saturation of 90% on room air and 94% with supplemental oxygen via nasal cannula at 2 L per minute and a tympanic temperature of 38.4 ºC.

On physical examination, the patient was oriented to person but disoriented to time and place, with confused speech; his skin and mucous membranes were pink, hydrated, and anicteric; he showed signs of respiratory distress with diminished vesicular breath sounds throughout the left lung field and normal breath sounds on the right, without adventitious sounds. Cardiac auscultation revealed rapid and irregular heart sounds without apparent murmurs. Abdominal examination revealed a non-tender, globular, and depressible abdomen with an irregular liver edge on palpation. There was no peripheral edema, and his extremities were warm with a capillary refill time of approximately 2 seconds. The arterial blood gas evaluation, without supplemental oxygen, showed hypoxemic respiratory failure (paO_2_ 59.2 mmHg), hypocapnia (paCO_2_ 28 mmHg), and partially compensated respiratory alkalemia (pH 7.505), along with hyperlactacidemia (lactate 2.49 mmol/L). From the laboratory data (Table 1), noteworthy findings included a complete blood count with neutrophilic leukocytosis (11.14 x 10^3^/μL and 73.3%) and elevated C-reactive protein; hyponatremia (129 mmol/L); and elevated NT-proBNP (1149 pg/mL), without other significant changes. The urine examination did not present findings compatible with infection.

**Table 1:** Laboratory findings on admission.

Complete Blood Count		Blood Biochemistry	
Red blood cells	4.5 x10^6^/μL	C-reactive protein	242 mg/L
Hemoglobin	12.0 g/dL	Lactate dehydrogenase	299 U/L
Hematocrit	40%	Total protein	6.4 g/dL
Platelet count	318 x 10^3^/μL	Albumin	3.8 g/dL
White blood cells	11.14 x 10^3^/μL	Blood urea	34 mg/dL
Neutrophils	73.3%	Creatinine	0.87 mg/dL
Lymphocytes	15.4%	Sodium	129 mmol/L
Monocytes	11.0%	Potassium	4.9 mmol/L
Eosinophils	0.3%	Chloride	98 mmol/L
Basophils	0%	Total bilirubin	1.2 mg/dL
-	-	Aspartate aminotransferase	49 U/L
Coagulation	-	Alanine aminotransferase	17 U/L
Activated partial thromboplastin time	29.5 seconds	Alkaline phosphatase	57 U/L
Prothrombin time	74%	N-terminal brain natriuretic	1149 pg/mL
International normalized ratio	1.17	peptide (NT-proBNP)	-
Arterial blood gas (no oxygen)		Urine	-
Potential of hydrogen (pH)	7.505	Potential of hydrogen (pH)	5.5
Partial pressure of arterial carbon dioxide (paCO_2_)	28 mmHg	Specific gravity	1.026
Partial pressure of arterial oxygen (paO_2_)	59.2 mmHg	Leukocytes	negative
Hydrogen carbonate	21.6 mmol/L	Protein	negative
Arterial oxygen saturation	90.8%	Glucose	negative
Lactate	2.49 mmol/L	Occult blood	negative
Pleural fluid biochemistry		Pleural fluid polymerase chain reaction (PCR) test	
Potential of hydrogen (pH)	7.5	Enterobacterales	Detected
Glucose	59 mg/dL	Klebsiella aerogenes	Not detected
Lactate dehydrogenase	257.9 U/L	Klebsiella oxytoca	Not detected
Albumin	3.2 g/dL	-	-
Total protein	5.1 g/dL	-	-
Pleural fluid cytological examination		Pleural fluid culture	
Nucleated cells	3211 cells/uL	Direct examination	Gram-negative bacilli
Polymorphonuclear	80%	Cultural examination	Klebsiella rhinoscleromatis
Mononuclear	20%	-	-
Erythrocytes	59310 cells/uL	Blood cultures	negative
Malignant cells	negative	-	-

**Figure 1:**
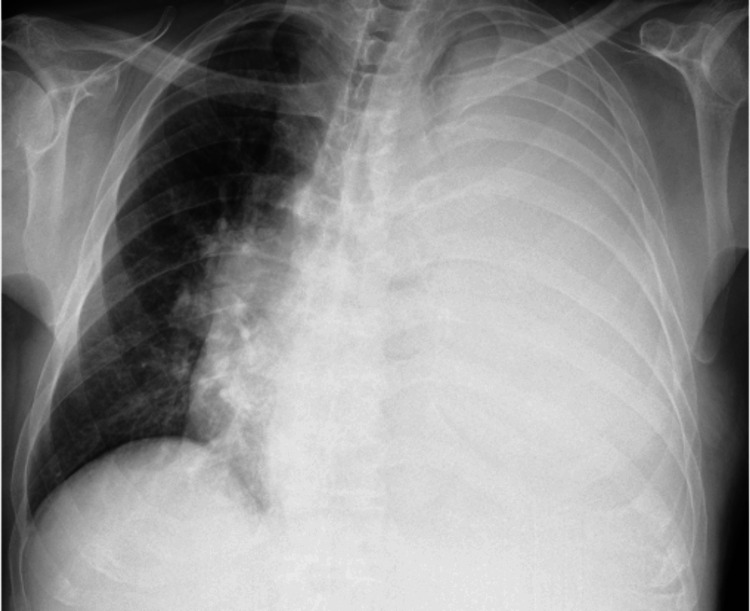
Chest radiograph (orthostatic position) showing a pleural effusion in the left lung field.

The electrocardiogram showed sinus tachycardia. The chest radiograph (Figure 1) revealed a complete white-out of the left hemithorax with tracheal shift to the contralateral side.

**Figure 2:**
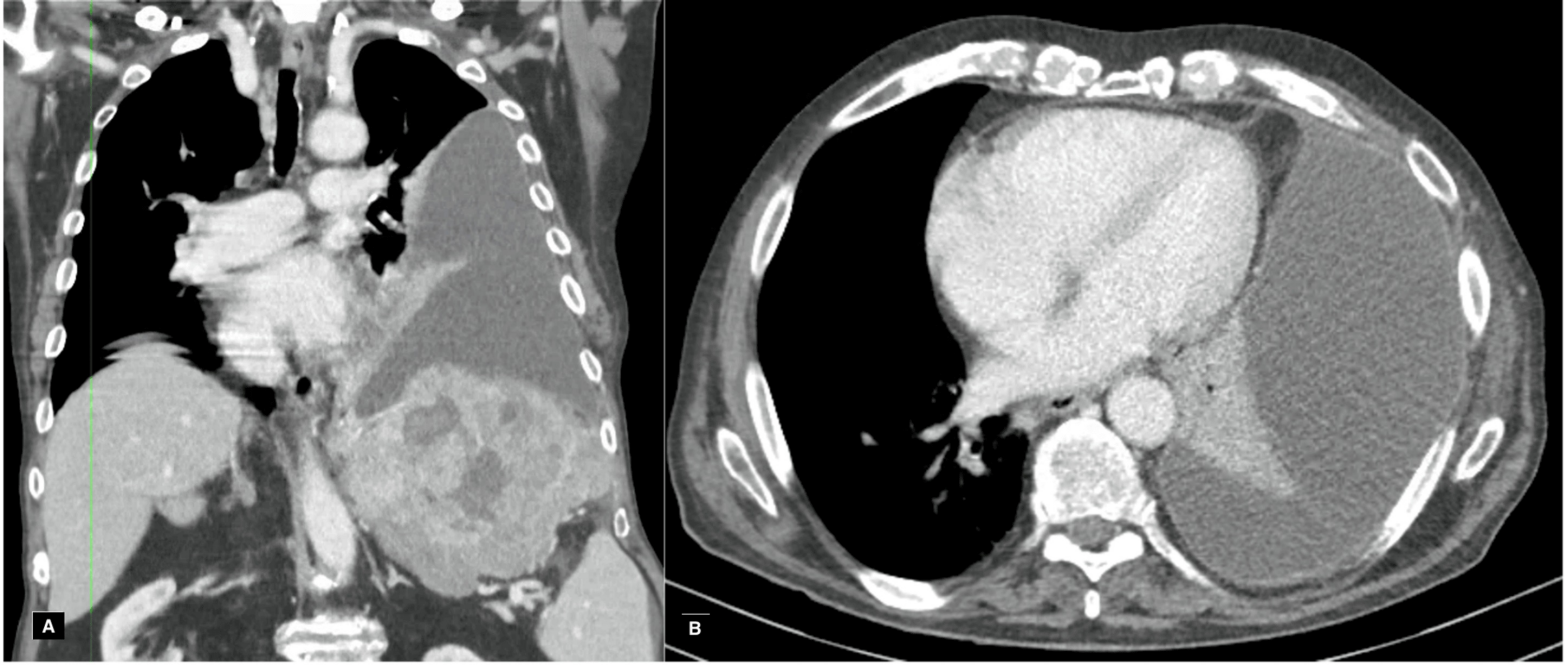
Chest and abdominal computed tomography showing pleural effusion in the left lung field, (A) coronal section and (B) axial section, with regular and diffuse pleural enhancement.

A chest computed tomography was performed for better characterization, which showed a large left pleural effusion with slight mediastinal shift and almost complete atelectasis of the left lung; no significant pleuroparenchymal changes on the right, specifically no consolidations or space-occupying lesions; and some mediastinal lymph node hypertrophy. The contrast-enhanced computed tomography (Figure 2) showed a regular and diffuse pleural enhancement and a large tumor lesion in the left lobe (192x120x129 mm) in a cirrhotic liver, with diaphragmatic and pleural invasion without ascites.

**Figure 3:**
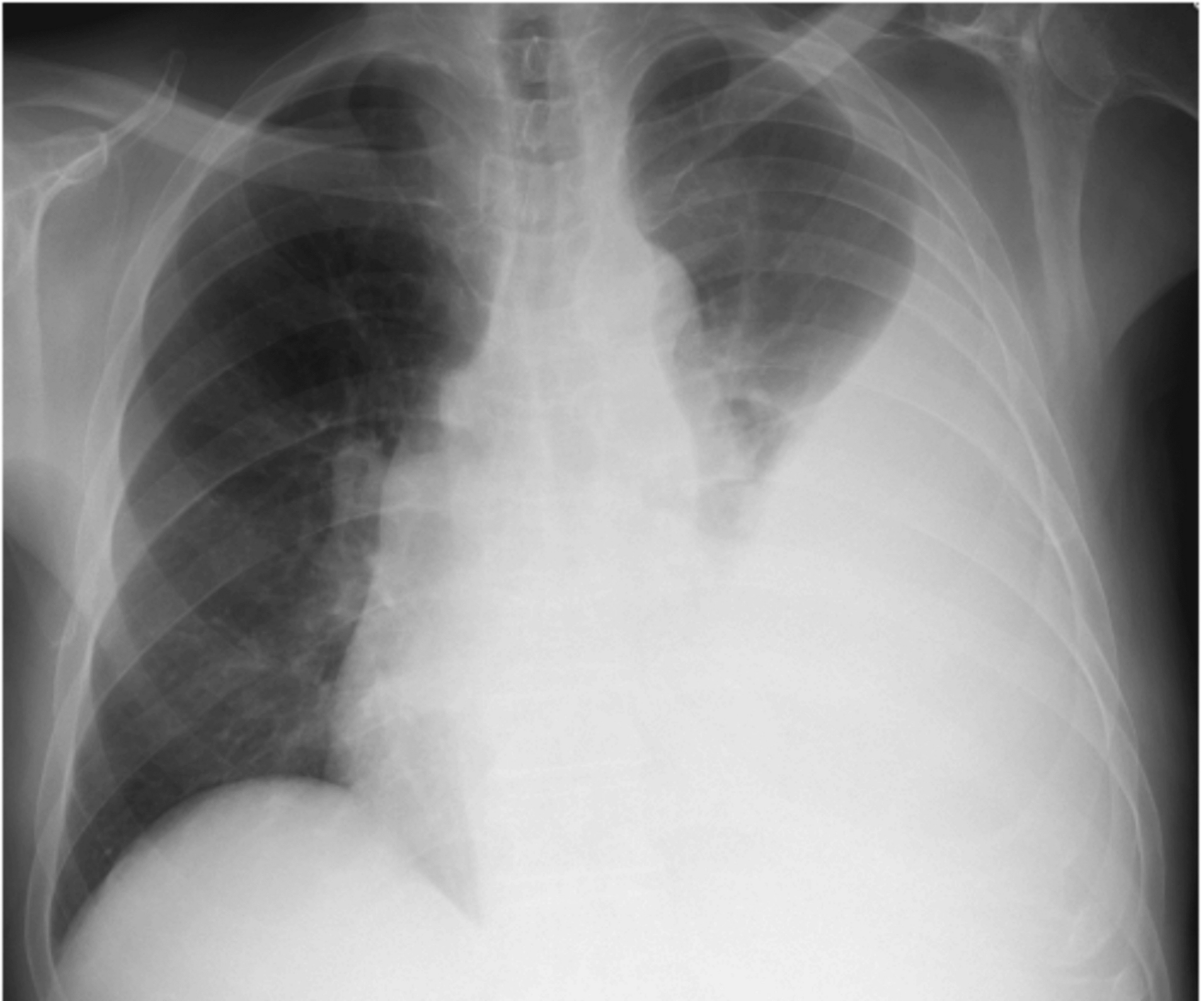
Chest radiograph (orthostatic position) showing a reduction in pleural effusion in the left lung field after thoracentesis.

Given the sepsis with a respiratory origin and a large-volume pleural effusion causing mediastinal shift and respiratory failure, thoracentesis was performed for both evacuation and diagnostic purposes. The thoracentesis allowed the removal of 1500 mL of sero-hematic fluid (Figure 3), and the biochemical analysis was consistent with an exudate according to Light's criteria (Table 1). The cytology exam of pleural fluid was negative for malignant cells and had moderate inflammatory characteristics, with a predominance of polymorphonuclear cells.

Empirical antibiotic therapy with ceftriaxone and azithromycin was initiated due to clinical suspicion of infection, and the patient was admitted to the internal medicine ward. Microorganisms of the *Enterobacterales* order were identified through polymerase chain reaction (PCR) testing of the pleural fluid, which was negative for *Klebsiella aerogenes* and *Klebsiella oxytoca*. Peripheral blood cultures were negative. The pleural fluid culture revealed growth of *Klebsiella rhinoscleromatis*, which was resistant only to ampicillin (Table 2). The antibiotic therapy was adjusted to include only intravenous ceftriaxone.

**Table 2:** Antibiotic sensitivity test for Klebsiella rhinoscleromatis.

Antibiotics	Interpretation
Ampicillin	Resistant
Ceftazidime	Sensitive
Cefotaxime	Sensitive
Ciprofloxacin	Sensitive
Cefuroxime	Sensitive
Ertapenem	Sensitive
Imipenem	Sensitive
Levofloxacin	Sensitive
Meropenem	Sensitive
Trimethoprim + Sulfamethoxazole	Sensitive
Amoxicillin + Clavulanic acid	Sensitive

**Figure 4:**
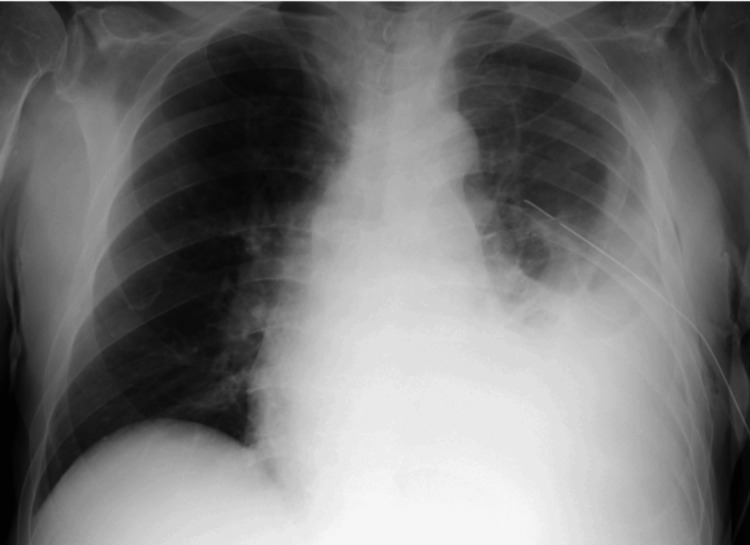
Chest radiograph (orthostatic position) showing the chest drain in the left lung field.

After reevaluation of the imaging, a worsening of the pleural effusion was observed, along with concurrent clinical deterioration, leading to the decision to place a chest drain (Figure 4), and again pleural fluid was collected. The new culture of the fluid did not reveal any microorganism growth, and antibiotic therapy was discontinued after 10 days of treatment.

Despite the resolution of the infectious condition and the measures taken, after 30 days of hospitalization, the patient's clinical condition worsened, culminating in multiple organ failure, and the patient ultimately died in this context.

## Discussion

*Klebsiella pneumoniae* subsp. *rhinoscleromatis* is a microorganism that typically causes chronic granulomatous infection of the upper airways, known as rhinoscleroma [5,6], first described by Von Frisch in 1882 [5,7]. Although rare worldwide, this pathology is endemic in the Middle East, Central and South America, and Eastern Europe [8,9]. Humans are the only host for this microorganism [8], which is more prevalent in rural areas with poor socioeconomic conditions, predominantly affecting women in their second and third decades of life [5,7]. The nose is affected in approximately 95%-100% of cases, but other regions, such as the trachea and bronchi, can also be involved [5]. Lower respiratory tract involvement without nasal changes is rare [7,10]. Thus, the presentation of infection by *Klebsiella pneumoniae* subsp. *rhinoscleromatis* as pneumonia is rare, and infections by this agent are frequently chronic, with life-threatening infections rarely reported [10,11]. Although uncommon, it is documented in the literature that this agent can colonize and cause lower respiratory tract infections, including severe pneumonia and sepsis [10,11]. It can also cause infections in other locations, leading to liver and prostate abscesses, meningitis, or endophthalmitis [12].

Considering the epidemiological data, despite this agent being rare in this region and community, the described patient had the main risk factor for *Klebsiella* infection, which is alcohol use disorder, contributing to malnutrition and loss of respiratory tract barriers [13]. Recognizing this etiologic agent is complex, given that the incidence in non-endemic areas is extremely low [9]. The diagnosis of rhinoscleroma is made through a biopsy or cultural examination [14]. In the described case, infectious pleural effusion was diagnosed based on the clinical presentation of fever, dyspnea, elevated inflammatory markers, and the computed tomography findings, which revealed regular and diffuse pleural enhancement compatible with an infectious-type effusion. This led to the initiation of empirical antibiotic therapy. The presence of *Enterobacterales* was detected later through pleural fluid PCR testing, and the definitive agent was then isolated in the culture of the fluid.

There are no studies determining the ideal therapy [15,16], as susceptibility to antibiotic therapy can be very variable, and combined therapy may be necessary [9]. Therapeutic options include third-generation cephalosporins and quinolones as monotherapy [15,16]. The described patient falls into this group, as he received third-generation cephalosporin (ceftriaxone) therapy from the beginning of treatment, later confirming the agent's susceptibility to this drug. Despite the prescription of targeted antibiotic therapy, the mortality rate for pneumonia caused by *Klebsiella pneumoniae* is approximately 50% [1], with a worse prognosis in the elderly and immunocompromised patients [10,13].

The sensitivity of the cytological examination of pleural fluid for detecting neoplastic cells is approximately 46% overall, although it can vary depending on the type of neoplasm [17]. No neoplastic cells were found in the pleural fluid of the described patient. However, given the large size of the tumor lesion, with imaging evidence of diaphragmatic and pleural invasion, the pleural effusion was assumed to be malignant in the context of the hepatic neoplasm.

In this case, the outcome was unfavorable, even after the resolution of the infection and the placement of the chest drain. The patient had been diagnosed with hepatocellular carcinoma in a cirrhotic liver at an advanced stage, with a poor prognosis and an expected survival of six to eight months, or approximately 25% after one year [18]. The poor clinical progression, which ultimately led to the patient's death, was attributed to this neoplastic context.

## Conclusions

*Klebsiella pneumoniae* subsp. *rhinoscleromatis* was initially described as a cause of chronic, non-lethal infection of the nasal region, called rhinoscleroma; however, it can also cause life-threatening infections such as sepsis from pneumonia. There are endemic areas, making diagnosis more difficult in other locations, but this type of agent is increasingly being isolated in immunocompromised patients outside these regions. There is no ideal therapy for severe infections, and there are various factors of virulence and antibiotic resistance, making the consideration of the diagnosis and early detection of the agent crucial in order to avoid unfavorable outcomes in patients with poor prognostic factors.

To the best of our knowledge, this is the first report of a patient with extensive malignant pleural effusion with infection caused by *Klebsiella pneumoniae* subsp. *rhinoscleromatis* in the English-language literature, making this case highly significant for reporting.
